# Economic and Humanistic Burden of Moderate and Severe Hemophilia A and B in Spain: Real-World Evidence Insights from the CHESS II Study

**DOI:** 10.36469/001c.92369

**Published:** 2024-05-06

**Authors:** Carmen Peral, Alfonso De Lossada Juste, Nadia Lwoff, Nataly Espinoza-Cámac, Miguel Ángel Casado, Tom Burke, Jose Alvir, Sheena Thakkar, Enrico Ferri Grazzi

**Affiliations:** 1 Pfizer S.L.U., Madrid, Spain; 2 Pharmacoeconomics and Health Outcomes Research Iberia (Spain); 3 HCD Economics, Daresbury, UK; 4 Faculty of Health and Social Care University of Chester, Chester, UK; 5 Pfizer Inc., New York, New York, USA; 6 Pfizer Inc., New York, NY, USA

**Keywords:** CHESS II, hemophilia A, hemophilia B, cost, moderate, severe

## Abstract

**Background:** Hemophilia is a congenital disorder characterized by deficiency or absence of clotting factor VIII in hemophilia A (HA) or clotting factor IX in hemophilia B (HB), resulting in frequent, repeated, and prolonged spontaneous or traumatic bleeding into joints or soft tissue. Severity is classified by the patient’s baseline level of clotting factor activity as mild (>5%-40%), moderate (1%-5%), or severe (<1%). In Spain, there is limited information on the societal economic burden of disease.

**Objective:** To estimate the economic and humanistic burden of disease in adult patients with non-inhibitor moderate and severe HA and HB in Spain.

**Methods:** Spanish data from the CHESS II study (2018-2020) on patients’ clinical characteristics, health-related quality of life (HRQoL) and hemophilia-related healthcare resource utilization were analyzed. Economic burden was determined by estimating condition-related annual per-patient direct (medical and nonmedical) and indirect costs, stratified according to hemophilia type and severity and presented as 2022 Euros. HRQoL was assessed via the EQ-5D-5L.

**Results:** Of 341 patients in the Spanish CHESS II cohort, 288 patients met the inclusion criteria: 181 had HA (37% [n = 66] moderate and 63% [n=115] severe) and 107 had HB (26% [n = 28] moderate and 74% [n = 79] severe). Mean annual direct cost was higher in HB than in HA, and higher in severe than in moderate patients, resulting in an annual cost/patient of €17 251 (moderate HA), €17 796 (moderate HB), €116 767 (severe HA) and €206 996 (severe HB). The main direct cost component in all groups except moderate HA was factor replacement therapy. Mean per-patient indirect cost was €4089 (moderate HA), €797 (moderate HB), €8633 (severe HA) and €8049 (severe HB). Finally, the mean total cost (direct and indirect) for moderate and severe patients were €91 017 (HA) and €163 924 (HB). EQ-5D-5L [SD] scores were lower in patients with severe HA (0.77 [0.18]) and severe HB (0.70 [0.22]) compared with patients with moderate HA (0.81 [0.15]) and moderate HB (0.86 [0.17]).

**Conclusions:** Independently of the type of hemophilia, greater condition severity was associated with increased costs and a decrease in HRQoL.

## INTRODUCTION

Hemophilia is an X-linked, recessive, congenital disorder characterized by a deficiency or absence of clotting factor VIII (FVIII) in the case of hemophilia A (HA) or clotting factor IX (FIX) in the case of hemophilia B (HB), which may lead to spontaneous acute bleeding or prolonged traumatic bleeding events.^1^ Depending on the clotting factor activity level (reported as percentage of normal activity), the severity of hemophilia is classified as mild (40% to >5%), moderate (5% to 1%) or severe (<1%).^2,3^

Hemophilia mainly affects males, with HA being more prevalent than HB.^2,3^ According to data from the World Federation of Hemophilia 2021 Global Survey (covering 118 countries), 185 318 patients with HA and 37 998 with HB were identified worldwide.^4^ The global prevalence, considering incidence and mortality rates, was estimated at 17.1 cases of HA and 3.8 cases of HB per 100 000 males, with prevalence of severe condition estimated as 3.8 for HA and 1.1 for HB.^4^ In 2010, Spain had patient number estimates of a total of 2595 (86.7%) HA and 398 (13.3%) HB patients.^5^ In 2017, considering the ratios of HA to HB in the published data on hemophilia in Spain (HA/HB ratios of 6.5:1^6^ and 5:1,^7^ respectively), the number of patients with HB reported was 317 cases.^8^ The publication on Spanish hemophilia epidemiology from a registry in 2013, which included patients with moderate and severe hemophilia identified that the proportion of severe disease (HA, 80.8%; HB, 60.6%) was higher than moderate disease (HA, 19.2%; HB, 39.4%).^7^

The characteristic symptom of hemophilia is the manifestation of bleeding episodes; bleeds that occur at the intra-articular and muscular level, can lead to chronic inflammation, causing hemophilic arthropathy, generating long-term disability.^9,10^ Bleeding occurring in the central nervous system can result in severe complications leading to death.^9^ The clinical burden for people with hemophilia is, therefore, persistent and lifelong.^11,12^ Both recurrent bleeding episodes and joint damage are also associated with increased healthcare resource utilization; the presence and extent of joint damage and bleeding has been associated with a significant socioeconomic burden in patients with severe hemophilia in terms of healthcare resource utilization, work productivity, daily functioning, and overall health-related quality of life (HRQoL).^13–15^

An improvement in treatment-related outcomes would help reduce the burden of disease. The standard of care for the patient includes prophylaxis and on-demand treatment with clotting factor replacement therapies (CFRT) or emicizumab (a monoclonal antibody indicated for routine prophylaxis of bleeding episodes in patients with HA).^9,16,17^ For prophylaxis with CFRT, standard half-life (SHL) intravenous infusion products (administered 2-3 times per week) are available; in addition, extended half-life (EHL) products have been developed. These EHL CFRTs reduce the frequency of dosing to 1-2 times per week, which may improve adherence and patient acceptance of prophylaxis.^9,16,17^ On the other hand, it is possible that the patient receiving CFRT may develop inhibitors (IgG antibodies) that neutralize CFRT, rendering treatment ineffective. Diagnosis and quantification of inhibitors should be performed in the laboratory when the presence of active inhibitors is suspected (ie, when a patient who normally responds to CFRT fails to respond.^9^

The available real-world evidence to establish optimal treatment strategies and utilization of healthcare resources in Spain is limited. In this regard, the aim of this study was to describe the sociodemographic and clinical characteristics identifying the economic and humanistic burden, from a social perspective, of adult patients with moderate and severe HA and HB in Spain, without inhibitor diagnosis, based on data from the Cost of Hemophilia in Europe: A Socioeconomic Survey II (CHESS II) study.

## METHODS

Data for this analysis were drawn from the CHESS II study, a cross-sectional, retrospective, burden-of-illness study of 1337 men (≥18 years) with hereditary HA and HB of any severity from 8 European countries (Spain, France, Germany, Italy, United Kingdom, Denmark, Netherlands, and Romania); data were collected between November 2018 and October 2020.^18^ The design and methodology of the CHESS II study have been described in previous publications.^19,20^ The data collected in the CHESS study consisted of retrospective collection of patient data in the 12 months prior to the visit to the treating physician. In brief, data were provided by the physician (retrospective data from medical records) and by the patient (using the Patient Participation and Public Engagement Questionnaire [PPIE]). The physician entered data into an electronic case record form (CRF), while the patient filled out the PPIE. The CRFs had to be validated by the investigating physician or an authorized staff member to certify that the data contained in the CRFs had been correctly recorded. All data in the CRFs were anonymized and coded prior to analysis. Ethics approval was granted by the Research Ethics Subcommittee of the Faculty of Health and Social Care, University of Chester (UK) within the study, and all patients or their legal representatives provided signed informed consent to participate in the study.^18^ This analysis included people with moderate or severe HA or HB, not diagnosed with inhibitor, who were living in Spain at the time of data collection.

### Patient Sociodemographic and Clinical Characteristics

Patient sociodemographic characteristics (education, home circumstances, and employment status) and clinical characteristics (age, weight, body mass index, baseline treatment patterns, and type of CFRT treatment used) were identified. Sociodemographic characteristics were reported by the patient in the PPIE questionnaire and clinical characteristics were extracted by the treating physician from the patients’ medical records.

### Clinical Outcomes

The main clinical outcomes included annual bleeding rate (ABR), target joints, problem joints, level of chronic pain, hospital admissions, and joint surgeries. ABR was calculated using the number of bleeding events experienced in the 12 months immediately prior to data collection.^20^ A target joint is defined as a joint in which at least 3 spontaneous bleeds have occurred within a consecutive 6-month period.^2^ Where 2 or fewer bleeds have occurred into the joint within a consecutive 12-month period, the joint is no longer considered a target joint.^2^ The “problem joint” metric was developed in an exercise based on published expert consensus and was designed as a patient-relevant outcome for use in clinical practice and analysis of hemophilia.^21^ A problem joint is defined as a joint affected by chronic pain and/or limited range of motion due to compromised joint integrity (ie, suffering from chronic synovitis and/or hemophilic arthropathy), with or without recurrent bleeding.^21,22^ The level of hemophilia-related chronic pain was reported using a 1-to-4 scale (“no pain” to “severe pain”) defined based on the use of analgesics and functional deficit. The absence of pain included no functional deficit and non-use of analgesic (except in case of acute hemarthrosis). Mild pain included a functional status where pain did not interfere with occupation or activities of daily living (ADL) and might require occasional non-narcotic analgesic. Moderate pain included a functional state where the pain partially or occasionally interfered with occupation or ADL and considered the use of non-narcotic medications. Severe pain was considered a functional state in which pain interfered with occupation or ADL and required frequent use of non-narcotic and narcotic medications.

### Healthcare Resource Use and Costs

Economic outcomes included direct (medical and nonmedical) and indirect hemophilia-related costs in the 12 months prior to data collection. A societal perspective was considered. Direct medical costs were obtained from physician-reported health resource use through the CRF incorporating several cost components. The CFRT consumption (SHL, EHL, plasma-derived FVIII, and plasma-derived FIX), specialist consultations, laboratory tests, diagnostics, and hospitalizations (due to bleeding episodes and joint surgical procedures) were included. Hemophilia-related hospital admissions included information on length of stay (day ward or ICU), both when due to a bleeding event or to a surgical joint procedure. The types of procedures included were arthrocentesis, arthrodesis, arthroplasty, arthroscopy, and synovectomy.^9,18,23^ In the case of direct nonmedical and indirect costs, resource consumption was obtained from the patient sample completing the PPIE questionnaire, whose data were extrapolated for the whole cohort. Nonmedical costs included patient-reported hemophilia-related expenses, such as driving expenses, formal care, out-of-pocket expenditure on medicines (over-the-counter or physician-prescribed), health devices and/or home alterations, additional alternative therapies as occupational therapist, swimming or yoga, and transfer payments (eg, disability allowance). Indirect costs included the patient’s work productivity loss due to hemophilia and the loss of earnings by an informal caregiver.

Direct and indirect costs were calculated by first taking the mean quantity of the resource used and then multiplying that mean value by the source unit cost of the resource (**Table S1**). Each CFRT acquisition cost was calculated on the basis of the ex-factory price published by the General Council of Official Associations of Pharmacists (BotPlus), applying the deductions established by the Royal Decree-Law 8/2010 for Spanish National Health System.^24,25^ Unit costs of each resource consumed was obtained from the eSalud database and Spanish data sources (other unit costs were sourced from the literature).^18,19,26,27^ For the calculation of work productivity loss and informal caregiver cost, the labor costs (€7.82/hour)^28^ and the minimum inter-professional salary (€16.35/hour)^29^ were applied respectively. All costs were expressed in euros valued for the year 2022 (€, 2022) (**Table S1**).

### Health-Related Quality of Life

The assessment of humanistic outcomes was included in the PPIE and was based on the responses to the EQ-5D-5L^30^ questionnaire (the index score was calculated using the EuroQol value set for Spain). Using the EQ-5D-5L questionnaire, 5 dimensions of health status (mobility, self-care, usual activities, pain/discomfort and anxiety/depression) were assessed at 5 levels of severity (no problems, mild problems, moderate problems, severe problems, or extreme problems).^30^ According to the patients’ responses to the dimensions, the EQ-5D-5L profiles or health states were determined. Subsequently, the EQ-5D-5L index (utility values) were calculated for the health states according to the Spanish valuation study estimates,^31^ which were reported on a scale between 0 and 1, where death has a value of 0 and perfect health a value of 1. However, according to the valuation studies methodology based on time trade-off, negative values representing states worse than death were feasible. In addition, patients reported their satisfaction with the hemophilia healthcare received on a scale ranging from 1 (not at all satisfied) to 10 (very satisfied).

## RESULTS

### Patient Sociodemographic And Clinical Characteristics

Of the total 341 Spanish patients in the CHESS II data set, the cohort that met the inclusion criteria for the analysis (adult patients with moderate or severe HA and HB, with no current inhibitor diagnosis) consisted of 288 patients. The sociodemographic and clinical characteristics of the patients are presented in **Table 1**. Patients with moderate or severe HA (n = 181; moderate [n = 66], severe [n = 115]) had a mean age of 40.55 years (SD, 14.93). Patients with moderate or severe HB (n = 107; moderate [n = 28], severe [n = 79]) had a mean age (SD) of 40.56 (14.49) years. Mean (SD) weight was 75.30 (9.29) kg and 76.50 (10.54) kg for the HA and HB cohort, respectively. The average body mass index (BMI) result was >25 kg/m^2^, between 24.79 (HA) and 24.71 (HB) kg/m^2^. In relation to education, home circumstances, and employment status, of the patients who met the inclusion criteria (n = 288), less than 35% (HA, n = 53; HB, n = 47) had completed college or had an advanced degree, 58% (HA, n = 114; HB, n = 53) had not completed college, and slightly more than 63% (HA, n = 110, HB, n = 70) were employed either part-time, full-time, or self-employed. Likewise, the three most frequent comorbidities among the total number of patients meeting the inclusion criteria were anxiety in 22% (HA, n = 45; HB, n = 17), osteoarthritis in 12% (HA, n = 26; HB, n = 9), and anemia in 10% (HA, n = 18; HB, n = 11). Regarding treatment strategy, there were no records (NR, not reported) of receiving prophylaxis in patients with moderate hemophilia (HA, n = NR; HB, n = NR). Prophylaxis (primary and secondary) was more frequently reported in patients with severe hemophilia, representing 41% (HA, n = 58; HB, n = 59) of the total number of patients meeting the inclusion criteria. The on-demand treatment strategy accounted for 38% (HA, n = 82; HB, n = 27) of the total group (N = 288).

**Table 1.** Sociodemographic and Clinical Characteristics of Patients with Hemophilia A and Hemophilia B in Spain

**Parameter**	**Hemophilia A**			**Hemophilia B**		
	**Moderate (n = 66)**	**Severe** **(n=115)**	**Moderate and Severe (n=181)**	**Moderate (n = 28)**	**Severe** **(n = 79)**	**Moderate and Severe (n = 107)**
General characteristics, mean (SD)						
Age, y	40.77 (16.08)	40.43 (14.29)	40.55 (14.93)	40.50 (13.40)	40.58 (14.93)	40.56 (14.49)
Weight, kg	74.79 (9.71)	75.6 (9.07)	75.30 (9.29)	77.61 (11.78)	76.1 (10.12)	76.50 (10.54)
Body mass index, kg/m2	24.63 (2.65)	24.88 (2.57)	24.79 (2.60)	24.65 (2.89)	24.73 (3.36)	24.71 (3.23)
Education, n (%)						
No schooling completed	2 (3.03)	NR	2 (1.10)	NR	NR	NR
No college	36 (54.55)	78 (67.83)	114 (62.98)	13 (46.43)	40 (50.63)	53 (49.53)
College or advanced degree	20 (30.30)	33 (28.70)	53 (29.28)	11 (39.29)	36 (45.57)	47 (43.93)
Other	8 (12.12)	4 (3.48)	12 (6.63)	4 (14.29)	3 (3.8)	7 (6.54)
Home circumstances, n (%)						
Lives alone	13 (19.70)	18 (15.65)	31 (17.13)	6 (21.43)	8 (10.13)	14 (13.08)
Lives with family/friends	36 (54.55)	74 (64.35)	110 (60.77)	7 (25.00)	44 (55.70)	51 (47.66)
Lives with partner	8 (12.12)	19 (16.52)	27 (14.92)	8 (28.57)	21 (26.58)	29 (27.10)
Nursing home	NR	NR	NR	1 (3.57)	1 (1.27)	2 (1.87)
Don’t know/other	9 (13.64)	4 (3.48)	13 (7.18)	6 (21.43)	5 (6.33)	11 (10.28)
Employment status, n (%)						
Full-time employed	25 (37.88)	41 (35.65)	66 (36.46)	12 (42.86)	37 (46.84)	49 (45.79)
Part-time employed^a^	8 (12.12)	27 (23.48)	35 (19.34)	3 (10.71)	9 (11.39)	12 (11.21)
Self-employed	1 (1.52)	8 (6.96)	9 (4.97)	3 (10.71)	6 (7.59)	9 (8.41)
Student	7 (10.61)	10 (8.70)	17 (9.39)	3 (10.71)	9 (11.39)	12 (11.21)
Retired	8 (12.12)	11 (9.57)	19 (10.50)	3 (10.71)	6 (7.59)	9 (8.41)
Unemployed	4 (6.06)	3 (2.61)	7 (3.87)	NR	6 (7.59)	6 (5.61)
Other	13 (19.70)	15 (13.04)	28 (15.47)	4 (14.29)	6 (7.59)	10 (9.35)
Comorbidities, n (%)						
Anemia	6 (9.09)	12 (10.43)	18 (9.94)	3 (10.71)	8 (10.13)	11 (10.28)
Anxiety	19 (28.79)	26 (22.61)	45 (24.86)	1 (3.57)	16 (20.25)	17 (15.89)
Atrial fibrillation	1 (1.52)	3 (2.61)	4 (2.21)	1 (3.57)	1 (1.27)	2 (1.87)
Attention deficit disorder	NR	NR	NR	1 (3.57)	1 (1.27)	2 (1.87)
Gingivitis	5 (7.58)	5 (4.35)	10 (5.52)	1 (3.57)	5 (6.33)	6 (5.61)
Hepatitis C	3 (4.55)	8 (6.96)	11 (6.08)	1 (3.57)	1 (1.27)	2 (1.87)
HIV	1 (1.52)	6 (5.22)	7 (3.87)	NR	NR	NR
Myocardial infarction	2 (3.03)	1 (0.87)	3 (1.66)	1 (3.57)	2 (2.53)	3 (2.80)
Obesity	3 (4.55)	6 (5.22)	9 (4.97)	4 (14.29)	5 (6.33)	9 (8.41)
Osteoarthritis	10 (15.15)	16 (13.91)	26 (14.36)	3 (10.71)	6 (7.59)	9 (8.41)
Osteoporosis	5 (7.58)	10 (8.69)	15 (8.29)	NR	3 (3.80)	3 (2.80)
Stroke	NR	1 (0.87)	1 (0.55)	1 (3.57)	1 (1.27)	2 (1.87)
Type I diabetes	2 (3.03)	1 (0.87)	3 (1.66)	1 (3.57)	1 (1.27)	2 (1.87)
Type II diabetes mellitus	6 (9.09)	6 (5.22)	12 (6.63)	NR	4 (5.06)	4 (3.74)
None	21 (31.82)	50 (43.48)	71 (39.23)	12 (42.86)	36 (45.57)	48 (44.86)
Treatment strategy, n (%)						
No treatment received 12 mo prior^b^	41 (62.12)	NR	41 (22.65)	21 (75.00)	NR	21 (19.63)
Primary prophylaxis	NR	22 (19.13)	22 (12.15)	NR	48 (60.76)	48 (44.86)
Secondary prophylaxis	NR	36 (31.30)	36 (19.89)	NR	11 (13.92)	11 (10.28)
Primary on-demand	20 (30.30)	49 (42.61)	69 (38.12)	6 (21.43)	18 (22.78)	24 (22.43)
Secondary on-demand	5 (7.58)	8 (6.96)	13 (7.18)	1 (3.57)	2 (2.53)	3 (2.80)
Treatment class, n (%)						
EHL	4 (6.06)	16 (13.91)	20 (11.05)	1 (3.57)	23 (29.11)	24 (22.43)
Plasma-derived	2 (3.03)	16 (13.91)	18 (9.94)	2 (7.14)	30 (37.94)	32 (29.91)
SHL	58 (87.87)	82 (71.30)	140 (77.35)	25 (89.29)	26 (32.91%)	51 (47.66)

^a^Part-time defined as employed ≤30 hours/week.

^b^No treatment was reported/needed in the 12 months immediately preceding data collection.

Abbreviations: EHL, extended half-life; NR, none reported; SHL, standard half-life.

### Clinical Outcomes

A higher mean (SD) ABR was observed in severe [HA, 4.36 (9.38); HB, 4.57 (6.44)] compared with moderate [HA, 2.77 (3.57); HB, 3.64 (4.27)] hemophilia. The mean (SD) annual number of bleeding-related hospitalizations in the total sample was 0.68 (0.92) for moderate and severe HA and 0.89 (0.86) for moderate and severe HB.

Of all target joints reported by physicians (HA, n = 84; HB, n = 29), the knee was the most commonly affected joint with 24% (HA, n = 58; HB, n = 12), also coinciding as the joint most reported as a problem joint (25%) (HA, n = 48; HB, n = 25) by patients.

In terms of patients’ chronic pain, the majority of the total patients included reported mild to moderate pain (70%) (HA, n = 118; HB, n = 84). Of the 181 patients with HA, 32% reported mild pain (moderate, n = 24; severe, n = 34) and 33% reported moderate pain (moderate, n = 22; severe, n = 38). Among the 107 patients with HB, almost half (48%) (moderate n = 11; severe, n = 40) reported mild pain, followed by those reporting moderate pain (30%) (moderate, n = 7; severe, n = 26).

Further details of the clinical outcomes of patients with HA and HB in Spain are presented in **Table 2**.

**Table 2.** Clinical Outcomes of Patients with Hemophilia A and Hemophilia B in Spain

**Parameter**	**Hemophilia A**			**Hemophilia B**		
	**Moderate (n=66)**	**Severe (n=115)**	**Moderate and Severe (n=181)**	**Moderate (n=28)**	**Severe (n=79)**	**Moderate and Severe (n=107)**
Bleeding outcomes						
ABR, mean (SD)	2.77 (3.57)	4.36 (9.38)	3.78 (7.80)	3.64 (4.27)	4.57 (6.44)	4.33 (5.94)
Cause of bleeding (proportion), % (SD)						
Trauma-related bleeding events	59.14 (29.43)	58.70 (29.52)	58.86 (29.41)	67.86 (29.48)	60.03 (27.93)	62.11 (28.42)
Spontaneous bleeding events	40.86 (29.43)	41.30 (29.52)	41.14 (29.41)	32.14 (29.48)	39.97 (27.93)	37.89 (28.42)
Target joints, n (%)^a^	23 (34.85)	61 (53.04)	84 (46.41)	9 (32.14)	20 (25.32)	29 (27.10)
Distribution of target joints, n (%)						
Ankle	3 (4.55)	12 (10.43)	15 (8.29)	2 (7.14)	4 (5.06)	6 (5.61)
Elbow	2 (3.03)	12 (10.43)	14 (7.73)	3 (10.71)	5 (6.33)	8 (7.48)
Hip	6 (9.09)	13 (11.30)	19 (10.50)	1 (3.57)	7 (8.86)	8 (7.48)
Knee	14 (21.21)	44 (38.26)	58 (32.04)	3 (10.71)	9 (11.39)	12 (11.21)
Neck	1 (1.52)	1 (0.87)	2 (1.10)	2 (7.14)	1 (1.27)	3 (2.80)
Shoulder	4 (6.06)	7 (6.09)	11 (6.08)	3 (10.71)	2 (2.53)	5 (4.67)
Spine	1 (1.52)	1 (0.87)	2 (1.10)	NR	2 (2.53)	2 (1.87)
Wrist	1 (1.52)	6 (5.22)	7 (3.87)	1 (3.57)	NR	1 (0.93)
Problem joints, n (%) ^b^	31 (46.97)	55 (47.83)	86 (47.51)	8 (28.57)	38 (48.10)	46 (42.99)
Distribution of problem joints, n (%)						
Ankle	12 (18.18)	16 (13.91)	28 (15.47)	1 (3.57)	12 (15.19)	13 (12.15)
Elbow	2 (3.03)	9 (7.83)	11 (6.08)	0 (0.00)	5 (6.33)	5 (4.67)
Hip	9 (13.64)	10 (8.70)	19 (10.50)	NR	7 (8.86)	7 (6.54)
Knee	17 (25.76)	31 (26.96)	48 (26.52)	3 (10.71)	22 (27.85)	25 (23.36)
Neck	NR	NR	NR	NR	NR	NR
Shoulder	2 (3.03)	9 (7.83)	11 (6.08)	2 (7.14)	4 (5.06)	6 (5.61)
Spine	4 (6.06)	3 (2.61)	7 (3.87)	1 (3.57)	NR	1 (0.93)
Wrist	4 (6.06)	6 (5.22)	10 (5.52)	1 (3.57)	4 (5.06)	5 (4.67)
Joint procedures (in prior 12 months), n (%)	10 (15.15)	29 (25.22)	39 (21.55)	1 (3.57)	5 (6.33)	6 (5.61)
Distribution of joint procedures, n (%)						
Ankle	2 (3.03)	NR	2 (1.10)	NR	1 (1.27)	1 (0.93)
Elbow	NR	1 (0.87)	1 (0.55)	NR	1 (1.27)	1 (0.93)
Hip	1 (1.52)	5 (4.35)	6 (3.31)	NR	1 (1.27)	1 (0.93)
Knee	6 (9.09)	26 (22.61)	32 (17.68)	NR	1 (1.27)	1 (0.93)
Neck	NR	NR	NR	NR	1 (1.27)	1 (0.93)
Shoulder	2 (3.03)	3 (2.61)	5 (2.76)	1 (3.57)	NR	1 (0.93)
Spine	NR	NR	NR	NR	NR	NR
Wrist	1 (1.52)	NR	1 (0.55)	NR	NR	NR
Surgical intervention type, n (%)						
Arthrocentesis	9 (13.64)	24 (20.87)	33 (18.23)	NR	6 (7.59)	6 (5.61)
Arthroscopy	6 (9.09)	9 (7.83)	15 (8.29)	NR	3 (3.80)	3 (2.80)
Arthrodesis	2 (3.03)	5 (4.35)	7 (3.87)	NR	NR	NR
Synovectomy	1 (1.52)	3 (2.61)	4 (2.21)	1 (3.57)	2 (2.53)	3 (2.80)
Arthroplasty	2 (3.03)	2 (1.74)	4 (2.21)	NR	1 (1.27)	1 (0.93)
No. of joint procedures, mean (SD; n)						
Total sample	0.58 (2.17; 66)	0.63 (1.76; 115)	0.61 (1.91; 181)	0.04 (0.19; 28)	0.15 (0.46; 79)	0.12 (0.41; 107)
Patients with ≥1 procedure	3.80 (4.52; 10)	2.48 (2.80; 29)	2.82 (3.31; 39)	1.00 (NA; 1)	1.33 (0.48; 9)	1.30 (0.48; 10)
No. of hospital admissions, mean (SD; n)						
Related to bleeding events ^c^						
Occurrences in the total sample	0.61 (0.70; 66)	0.72 (1.03; 115)	0.68 (0.92; 181)	0.50 (0.81; 28)	1.11 (0.81; 79)	0.89 (0.86; 107)
Occurrences in patients with ≥1 bleed-related hospitalization	1.21 (0.48; 33)	1.73 (0.89; 48)	1.52 (0.79; 81)	1.44 (0.73; 9)	1.36 (0.68; 36)	1.38 (0.68; 45)
Related to joint procedures ^d^						
Occurrences in the total sample	0.36 (1.35; 66)	0.39 (1.01; 115)	0.38 (1.14; 181)	0.04 (0.19; 28)	0.14 (0.42; 79)	0.11 (0.37; 107)
Occurrences in patients with ≥1 procedure-related ward stays	2.67 (2.83; 9)	1.88 (1.45; 24)	2.09 (1.91; 33)	1.00 (NA; 1)	1.22 (0.44; 9)	1.20 (0.42; 10)
Chronic pain level related to hemophilia, n (%)						
No pain	18 (27.27)	33 (28.70)	51 (28.18)	10 (35.71)	8 (10.13)	18 (16.82)
Mild pain	24 (36.36)	34 (29.57)	58 (32.04)	11 (39.29)	40 (50.63)	51 (47.66)
Moderate pain	22 (33.33)	38 (33.04)	60 (33.15)	7 (25.00)	26 (32.91)	33 (30.84)
Severe pain	2 (3.03)	10 (8.70)	12 (6.63)	NR	5 (6.33)	5 (4.67)

^a^Proportion of the sample that had at least 1 target joint.

^b^Proportion of the sample that had a surgical intervention in their joints in the past 12 months.

^c^Number of times a patient has required ward hospitalization (≥1 night) due to a bleeding event.

^d^Number of times a patient has required ward hospitalization (≥1 night) due to a joint procedure.

Abbreviations: ABR, annual bleed rate; IU, international unit; NA, not available due to sample size; NR: none reported.

### Healthcare Resource Use and Costs

Resource consumption for the management of HA and HB varied across condition severity (**Table 3**). In general, patients with severe hemophilia presented a higher mean number of specialist consultations than patients with moderate hemophilia during the 12 months prior to data collection. Among the specialties reported, visits to hematologists and specialist nurses were the most common. The mean (SD) annual number of visits (scheduled and unscheduled) to the treating hematologist was 8.73 (8.67) for moderate HA, 10.26 (7.59) for severe HA, 8.61 (9.94) for moderate HB, and 7.48 (5.29) for severe HB. The mean number of hemophilia nurse specialist visits, on the other hand, was 8.20 (10.20) for moderate HA, 9.90 (11.15) for severe HA, 8.36 (10.38) for moderate HB, and 10.82 (21.32) for severe HB.

**Table 3.** Resource Use and Direct Cost Components for Patients with Hemophilia A and Hemophilia B in Spain

**Healthcare Cost Components and Resource Use**	**Hemophilia A (HA)**			**Hemophilia B (HB)**		
	**Moderate (n=66)**	**Severe (n=115)**	**Moderate and Severe (n=181)**	**Moderate (n=28)**	**Severe (n=79)**	**Moderate and Severe (n=107)**
Specialist consultations per patient in the past 12 months, mean (SD)						
Treating hematologist visits	8.73 (8.67)	10.26 (7.59)	9.70 (8.01)	8.61 (9.94)	7.48 (5.29)	7.78 (6.78)
Nurse specialist visits	8.20 (10.20)	9.90 (11.15)	9.28 (10.82)	8.36 (10.38)	10.82 (21.32)	10.18 (19.06)
Dentistry	0.80 (1.11)	1.17 (0.58)	1.03 (0.72)	0.92 (2.01)	1.40 (0.74)	1.25 (1.22)
Diet and nutritional support	0.44 (0.54)	0.40 (0.33)	0.41 (0.38)	0.73 (0.96)	0.81 (0.90)	0.78 (0.92)
Emergency medicine	0.86 (0.36)	1.25 (0.82)	1.11 (0.70)	1.40 (0.83)	2.29 (1.58)	2.02 (1.43)
General practice	2.35 (1.18)	3.45 (3.20)	3.05 (2.67)	4.50 (2.42)	4.88 (4.29)	4.76 (3.98)
General surgery	0.23 (0.50)	0.26 (0.05)	0.25 (0.17)	0.80 (1.73)	0.66 (0.22)	0.71 (0.92)
Genetics	0.42 (0.46)	0.21 (0.08)	0.29 (0.18)	1.02 (1.07)	0.45 (0.27)	0.72 (0.61)
Another hematologist	3.64 (3.68)	1.11 (1.19)	2.03 (1.84)	9.79 (10.02)	4.21 (1.73)	6.88 (5.38)
Hepatology	0.17 (0.21)	0.26 (0.16)	0.23 (0.18)	0.60 (0.57)	0.66 (0.54)	0.64 (0.55)
Immunology	0.23 (0.32)	0.17 (0.13)	0.19 (0.18)	0.58 (0.77)	0.44 (0.33)	0.49 (0.49)
Infectious diseases	0.24 (0.11)	0.34 (0.14)	0.30 (0.13)	0.56 (0.42)	0.87 (0.45)	0.77 (0.44)
Orthopedics	0.85 (0.71)	1.59 (0.78)	1.32 (0.77)	1.18 (0.94)	2.25 (1.58)	1.96 (1.44)
Pain management	0.76 (0.71)	0.93 (0.25)	0.87 (0.37)	1.70 (1.86)	1.66 (0.57)	1.67 (1.08)
Physiatry/rehabilitation	0.58 (0.46)	1.29 (0.78)	1.03 (0.70)	1.04 (0.92)	1.91 (1.52)	1.68 (1.40)
Physiotherapy	2.74 (2.46)	4.39 (3.13)	3.79 (2.95)	6.54 (9.55)	8.22 (6.73)	7.67 (7.52)
Psychiatry	0.23 (0.14)	0.37 (0.13)	0.31 (0.13)	0.55 (0.45)	1.33 (0.40)	1.11 (0.41)
Psychology	1.32 (0.39)	1.53 (0.39)	1.45 (0.39)	3.79 (1.17)	3.90 (0.98)	3.85 (1.03)
Rheumatology	0.17 (0.11)	0.17 (0.05)	0.17 (0.07)	0.38 (0.31)	0.48 (0.22)	0.45 (0.25)
Urology	0.32 (0.11)	0.27 (0.34)	0.29 (0.28)	0.79 (0.42)	0.61 (0.75)	0.68 (0.68)
Lab tests per patient in the past 12 months, mean (SD)						
Biochemistry blood test	5.52 (4.43)	5.80 (4.38)	5.70 (4.39)	6.52 (7.08)	4.85 (3.32)	5.51 (4.57)
CD4 count	0.82 (0.50)	1.17 (1.39)	1.04 (1.16)	1.39 (0.92)	2.07 (1.67)	1.85 (1.55)
Chromogenic factor assay	1.70 (0.64)	2.48 (2.06)	2.19 (1.69)	3.14 (1.22)	4.26 (2.51)	3.90 (2.32)
Coagulation test	5.92 (4.50)	6.03 (4.33)	5.99 (4.37)	8.27 (8.28)	5.47 (3.44)	6.61 (5.12)
Computed tomography	0.55 (0.36)	1.04 (0.67)	0.86 (0.59)	0.77 (0.56)	1.10 (0.75)	1.02 (0.71)
HCV antibody	1.23 (0.79)	1.10 (1.56)	1.14 (1.36)	1.51 (1.32)	1.65 (1.73)	1.60 (1.66)
Hemoglobin	5.82 (4.64)	6.18 (4.41)	6.05 (4.47)	6.50 (5.79)	5.49 (3.31)	5.86 (4.08)
HIV antibody	1.15 (0.75)	1.10 (1.53)	1.12 (1.33)	1.38 (0.93)	1.59 (1.73)	1.51 (1.59)
Magnetic resonance imaging (MRI)	0.53 (0.29)	0.87 (0.48)	0.75 (0.43)	0.88 (0.46)	1.12 (0.68)	1.05 (0.63)
Mix test	1.73 (0.68)	2.23 (1.03)	2.04 (0.93)	3.16 (1.33)	4.04 (2.62)	3.74 (2.35)
One-stage factor assay	2.05 (0.93)	2.44 (1.23)	2.30 (1.15)	3.11 (1.54)	3.87 (2.40)	3.61 (2.21)
Ultrasonography	1.18 (0.93)	1.49 (1.71)	1.38 (1.50)	1.59 (1.33)	1.54 (1.46)	1.56 (1.46)
Urinalysis	2.20 (1.54)	2.18 (2.33)	2.19 (2.12)	2.67 (2.15)	2.33 (1.96)	2.45 (2.03)
X-ray	1.74 (1.29)	2.23 (1.49)	2.05 (1.44)	2.13 (1.18)	1.79 (1.16)	1.93 (1.17)
Hemophilia-related hospitalizations (n), mean (SD)						
No. hospitalizations due to any hemophilia-related reason	0.86 (1.20)	0.97 (1.23)	0.93 (1.22)	0.79 (1.17)	0.80 (1.32)	0.79 (1.28)
Bleed-related (in patients with ≥1)						
Day cases, occurrences	1.00 (0.00; 4)	1.00 (0.00; 3)	1.00 (0.00; 7)	1.33 (0.58; 3)	1.13 (0.35; 8)	1.18 (0.40; 11)
Ward stay, LOS (days)	4.76 (2.72; 29)	6.24 (4.64; 45)	5.66 (4.04; 74)	6.86 (4.10; 7)	5.46 (7.69;28)	5.74 (7.09; 35)
ICU stay, LOS (days)	1.33 (0.58; 3)	1.90 (0.99; 10)	1.77 (0.93; 13)	NR	1.00 (0.00; 2)	1.00 (0.00; 2)
Joint surgery–related (in patients with ≥1)						
Day cases, occurrences	1.33 (0.58; 3)	1.44 (1.01; 9)	1.42 (0.90; 12)	NR	1.00 (NA; 1)	1.00 (NA; 1)
Ward stay, LOS (days)	5.56 (5.22; 9)	5.58 (8.43; 24)	5.58 (7.61; 33)	3.00 (NA; 1)	6.33 (6.95; 9)	6.00 (6.63; 10)
Joint surgeries, mean (SD)						
Arthrocentesis	0.29 (0.92)	0.38 (0.91)	0.35 (0.92)	NR	0.08 (0.27)	0.06 (0.23)
Arthrodesis	0.05 (0.27)	0.07 (0.41)	0.06 (0.37)	NR	NR	NR
Arthroplasty	0.03 (0.17)	0.02 (0.13)	0.02 (0.15)	NR	0.01 (0.11)	0.01 (0.10)
Arthroscopy	0.17 (0.78)	0.09 (0.31)	0.12 (0.53)	NR	0.04 (0.19)	0.03 (0.17)
Synovectomy	0.05 (0.37)	0.07 (0.57)	0.06 (0.51)	0.04 (0.19)	0.03 (0.16)	0.03 (0.17)
CFRT per treatment class (IU), mean (SD)						
EHL	19 800 (14 033)	315 886 (284 559)	263 635 (281 696)	192 000 (NA)	389 080 (627 036)	380 511 (613 996)
Plasma-derived	92 100 (124 309)	173 467 (321 100)	163 894 (303 172)	10 734 (1604)	365 173 (397 331)	341 544 (393 790)
SHL	10 821 (37 533)	202 460 (322 190)	120 126 (261 804)	2700 (9991)	257 108 (193 554)	134 992 (188 800)
All treatment classes, mean	13 828 (41 935)	213 338 (317 214)	138 964 (270 003)	10 306 (37 571)	335 124 (427 448)	249 977 (394 098)

Abbreviations: EHL, extended half-life; ICU, intensive care unit; NA, not available due to sample size; NR, none reported; LOS, length of stay; SHL, standard half-life.

The mean annual consumption of EHL-CFRT per patient was estimated at 19 800 IU (moderate HA), 315 886 IU (severe HA), 192 000 IU (moderate HB) and 389 080 IU (severe HB). The mean annual consumption of SHL-CFRT per patient was estimated at 10 821 IU (moderate HA), 202 460 IU (severe HA), 2700 IU (moderate HB), and 257 108 IU (severe HB). The mean annual consumption of plasma-derived CFRT per patient was 92 100 (moderate HA), 173 467 (severe HA), 10 734 (moderate HB), and 365 173 (severe HB). Finally, the mean annual consumption of all classes (EHL, SHL, and plasma-derived) per patient was 13 828 (moderate HA), 213 338 (severe HA), 10 306 (moderate HB), and 335 124 (severe HB). The average consumption of all classes (EHL, SHL, and plasma derivatives) were the data used to determine the cost of CFRTs.

Resource consumption of the nonmedical cost components obtained from the PPIE population (**Table 4**) reflects a higher number of hemophilia care-related visits to the treatment center hospital, or pharmacy for patients with severe disease. These visits were for medication pick-up, examination results, blood tests, or anything else related to hemophilia care. Of the 288 patients included, 153 (HA, n = 97; HB, n = 56) completed the PPIE, but not all patients completed data corresponding to all direct nonmedical cost components or indirect cost components. The mean (SD) number of visits in the previous 12 months reported among patients who completed the PPIE questionnaire was 32.66 (78.49) for patients with severe HA (completed PPIE, n = 65) and 21.97 (27.45) for patients with severe HB (completed PPIE, n = 39). The need for a formal caregiver ranged from a mean of 4.25 hours per week per patient with severe HB (n = 4) to 5.00 hours per week in the case for the patient with moderate HA (n = 4). Within the same population, the mean (SD) requirement for informal care was 7.84 (7.78) hours per week for patients with moderate or severe HA (n = 38) and 13.61 (9.86) for patients with moderate or severe hemophilia B (n = 18), respectively.

**Table 4.** Direct Nonmedical and Indirect Cost Components for Patients With Hemophilia and Hemophilia B in Spain (PPIE Population Only)

	**Hemophilia A (HA)**			**Hemophilia B (HB)**		
	**Moderate (n=28)**	**Severe (n=69)**	**Moderate and Severe (n=97)**	**Moderate (n=11)**	**Severe (n=45)**	**Moderate and Severe (n=56)**
**Direct Nonmedical Cost Components (Direct Patient/System Cost)**						
Transport to treatment center^a^						
Distance in km, mean (SD)	10.11 (9.89; 27)	8.75 (9.57; 63)	9.16 (9.63; 90)	10.91 (16.87; 1)	13.07 (18.98; 42)	12.62 (18.43; 53)
Mean visits, n (SD)^b^	10.38 (8.33; 24)	32.66 (78.49; 65)	26.65 (67.81; 89)	7.73 (7.76; 11)	21.97 (27.45; 39)	18.84 (25.15; 50)
Patient care (formal)^a^						
Hours/week, mean (SD)	5.00 (10.00; 4)	4.29 (6.82; 14)	4.44 (7.30; 18)	NR	4.25 (4.35; 4)	4.25 (4.35; 4)
Hours/year, mean (extrapolated)	260.00	222.86	231.11	NR	221.00	221.00
Health devices/home alterations						
Health devices, n (%)^a^						
Brace	2 (7.14)	13 (18.84)	15 (15.46)	NR	3 (6.67)	3 (5.36)
Cane	5 (17.86)	14 (20.29)	19 (19.59)	1 (9.09)	7 (15.56)	8 (14.29)
Crutches	4 (14.29)	6 (8.70)	10 (10.3)	2 (18.18)	4 (8.89)	6 (10.71)
Wheelchair	NR	2 (2.90)	2 (2.06)		1 (2.22)	1 (1.79)
Home alterations, n (%)^a^						
Ramp	1 (3.57)	NR	1 (1.03)	NR	1 (2.22)	1 (1.79)
Stairlift	NR	2 (2.90)	2 (2.06)	NR	NR	NR
Walk-in shower	4 (14.29)	12 (17.39)	16 (16.49)	1 (9.09)	2 (4.44)	3 (5.36)
Alternative therapies (privately funded), mean No. of sessions^a^						
Chiropractor, mean (SD; n)	4.2 (3.5; 6)	3.7 (5.8; 11)	3.9 (5.0; 17)	12.0 (NA; 1)	8.0 (NA; 1)	10.0 (2.8; 2)
Massage/⁠acupuncture, mean (SD; n)	3.0 (2.6; 3)	4.4 (7.2; 8)	4.0 (6.1; 11)	NR	56.0 (62.2; 2)	56.0 (62.2; 2)
Nutritionist, mean (SD; n)	1.3 (1.0; 4)	5.3 (11.1; 12)	4.3 (9.7; 16)	2.0 (1.4; 2)	1.0 (NA; 1)	1.7 (1.2; 3)
Occupational therapy, mean (SD; n)	5.0 (7.1; 2)	2.7 (4.3; 6)	3.3 (4.7; 8)	24.0 (NA; 1)	NR	24.0 (NA; 1)
Reflexology, mean (SD; n)	2.0 (1.6; 5)	14.9 (33.3; 7)	9.5 (25.5; 12)	12.0 (NA; 1)	NR	12.0 (NA; 1)
Swimming/aerobics, mean (SD; n)	19.5 (18.6; 4)	27.7 (37.4; 18)	26.2 (34.6; 22)	100.0 (70.7; 2)	14.8 (7.5; 4)	43.2 (54.5; 6)
Physiotherapist, mean (SD; n)	5.5 (3.7; 11)	11.2 (10.4; 29)	9.7 (9.4; 40)	12.0 (11.3; 2)	12.3 (15.3; 20)	12.3 (14.8; 22)
Psychologist, mean (SD; n)	3.5 (4.9; 2)	6.2 (9.6; 16)	5.9 (9.2; 18)	NR	5.0 (3.7; 7)	5.0 (3.7; 7)
Yoga/Pilates, mean (SD; n)	11.8 (12.0; 4)	12.8 (13.8; 14)	12.6 (13.1; 18)	24.0 (0.0; 2)	14.5 (9.7; 4)	17.7 (9.0; 6)
Transfer payments, n (%)^a^						
Received in the 12 mo prior, n (%)	4 (14.29)	15 (21.74)	19 (19.59)	NR	8 (17.78)	8 (14.29)
Monthly amount received, mean (SD)	250.00 (187.08)	434.93 (332.93)	396.00 (313.12)	NR	363.75 (301.33)	363.75 (301.33)
**Indirect cost components (productivity and societal)**						
Patient care (informal)^a,c^						
Hours/week, mean (SD; n)	8.73 (7.90; 11)	7.48 (7.85; 27)	7.84 (7.78; 38)	10.00 (14.14; 2)	14.06 (9.75; 16)	13.61 (9.86; 18)
Hours/year (extrapolated), mean	455.07	390.11	408.91	521.43	733.26	709.72
Productivity loss^a,d^						
Hours missed (3 months), mean (SD; n)	14.00 (8.28; 8)	12.95 (7.06; 42)	13.12 (7.18; 50)	14.00 (7.66; 4)	19.16 (6.32; 38)	18.67 (6.53; 42)
Hours/year missed (extrapolated), mean	56.00	51.84	52.48	56.00	76.48	74.56
Inability to work/early retirement, n (%)	2 (7.4)	14 (20.3)	16 (16.7)	NR	7 (15.9)	7 (13.0)

^a^It is assumed that the PPIE population data represent the total sample; this value is used for the extrapolation of costs using the proportions indicated.

^b^Visits to the treatment center, hospital, or hospital pharmacy for any reason related to hemophilia care.

^c^The data were provided by the patient related to an unpaid caregiver (friend or family member).

^d^The data correspond to the number of hours lost in the last 3 months.

Abbreviations: NA, not available due to sample size; NR, none reported; PPIE, Patient Public Involvement Engagement.

**Figure 1.**
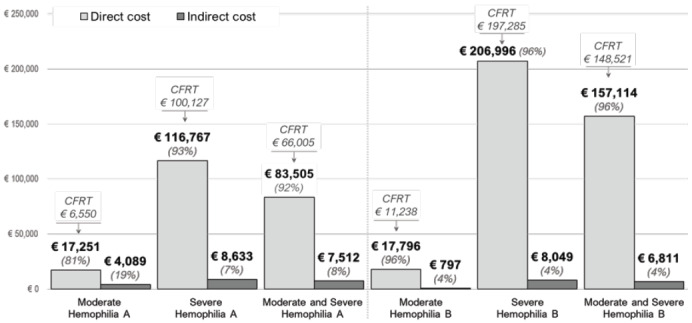
Annual Direct Costs per Patient with Hemophilia A and Hemophilia B in Spain Abbreviation: CFRT, clotting factor replacement therapies.

The economic burden increased substantially with increasing severity **(Table 5; Figure 1**). The mean annual per-patient direct cost was €83 505 for HA (€17 251 for moderate HA; €116 767 for severe HA) and €157 114 for HB (€17 796 for moderate HB; €206 996 for severe HB). According to the type of hemophilia, CFRT consumption represents 79% (€66 005) of the direct cost of HA and 95% (€148 521) of the direct cost of HB. The mean annual per-patient indirect cost ranged from €4089 (moderate HA) to €8049 (severe HB). The total annual per-patient cost (direct and indirect cost) was €21 340 to €125 400 for patients with moderate and severe HA, respectively; while in HB it ranged from €18 592 to €215 045 for moderate and severe condition, respectively. The average annual per-patient cost for HA and HB was €91 017 and €163 925, respectively. The average annual per-patient cost was €91 017 for HA and €163 924 for HB.

**Table 5.** Annual Direct and Indirect Cost Per Patient (€, 2022)

	**Hemophilia A**			**Hemophilia B**		
	**Moderate**	**Severe**	**Moderate and Severe**	**Moderate**	**Severe**	**Moderate and Severe**
Direct cost	**17 251.40**	**116 767.00**	**83 504.80**	**17 795.63**	**206 996.47**	**157 113.67**
Medical cost (only CFRT)	6549.92	100 127.14	66 005.06	11 238.11	197 285.05	148 521.24
Other medical cost^a^	8259.74	10 518.19	9905.50	5842.40	6523.67	6221.37
Nonmedical cost^b^	2441.74	6121.66	7594.24	715.11	3187.75	2371.06
Indirect cost	**4088.77**	**8633.27**	**7512.30**	**796.87**	**8048.88**	**6810.70**
Total cost	**21 340.17**	**125 400.27**	**91 017.11**	**18 592.50**	**215 045.34**	**163 924.37**

^a^Direct medical costs include visits to specialists, diagnostic tests, hospitalization, and procedures costs.

^b^Direct nonmedical costs include driving cost, formal care, health devices, home alterations, OTC medication (expenditure reported by patients), physician-prescribed medication (expenditure reported by patients), chiropractor (private) (extrapolated), massage/acupuncture (private) (extrapolated), nutritionist (private) (extrapolated), occupational therapist (extrapolated), reflexology (extrapolated), swimming/aerobics (extrapolated), physiotherapist (private) (extrapolated), psychologist (private) (extrapolated), yoga/Pilates (extrapolated), and transfer payments.

Abbreviations: CFRT, clotting factor replacement therapies; OTC, over-the-counter drugs.

### Health-Related Quality of Life

The mean (SD) EQ-5D-5L score reported by Spanish patients was 0.81 (0.15) for moderate HA, 0.77 (0.18) for severe HA, 0.86 (0.17) for moderate HB and 0.70 (0.22) for severe HB **(Table S2**).

## DISCUSSION

The CHESS II study aimed to provide a point estimate of the economic, social, and humanistic burden of hemophilia. To our knowledge, this is the first analysis to provide an estimate of the economic and humanistic burden of HA and HB focused on Spanish adult patients without inhibitor diagnosis, based on data obtained in the CHESS II study.

In this analysis, it was observed that the general characteristics (age, weight, and BMI) were similar between patients with HA and HB. Considering the mean age (41 years), more than half (58%) of the patients who met the inclusion criteria had no university education, just over a third (35%) had university or higher education, and more than half (63%) were employed. Regarding medical history, the most frequent comorbidities were anxiety, osteoarthritis, and anemia. In addition, variability in treatment strategies was observed (ie, patients in moderate grades [HA and HB] rarely received prophylaxis despite the apparent substantial disease burden), and in severe grades, prophylaxis was administered in less than half (41%) of patients. The on-demand strategy was used in more than a third of cases (38%). in alignment with the results presented in the analysis of the CHESS II study involving 5 European countries.^18^

In both types of hemophilia, the most frequently affected joint was the knee, a joint that also reported a higher number of surgical interventions in patients with severe HA. This reflects the potential benefit in carrying out early detection programs for arthropathy in this type of condition.^32^ A possible relationship was observed between increasing hemophilia severity and decreasing HRQoL as measured by the EQ-5D-5L. Lower utilities were reported for severe grades of hemophilia (HA, 0.77; HB, 0.70) compared with moderate grades of hemophilia (HA, 0.81; HB, 0.86). Likewise, the EQ-VAS assessment captured this decrease in HRQoL in patients with severe HA and HB, reporting a difference of 9 and 6 points, respectively. In other studies, decreased perception of quality of life was associated with increased joint problems,^22^ and also with the need for pain relief in hemophilic arthropathy.^33^

The estimation of direct and indirect costs showed that the severity of hemophilia is associated with a greater economic burden. In relation to the total cost, direct costs accounted for the largest proportion of the cost (92% for HA and 96% for HB). The average annual direct cost per patient with severe hemophilia was the highest (HA, €116 767; HB, €206 996), with the cost of CFRT representing 86% and 95% for HA and HB, respectively. The economic burden of CFRT use was in line with the previous analysis of the CHESS study in patients with severe hemophilia, conducted in 5 countries in Europe,^19^ which reflected that the economic burden of CFRT accounted for around 95% to 99% of the direct costs.

The study has the following limitations: first, the cost calculation methodology was performed using mean patient consumption data, which limits the ability to incorporate the variability (SD) of resource use collected in the CHESS II study, so the cost results lost this variability. However, this assumption would similarly affect all groups analyzed, since the objective was to determine costs between HA and HB, at the levels of moderate and severe severity.

For direct nonmedical and indirect costs, since data were not available for the total cohort, the mean per-patient cost was calculated for the PPIE sample, so that, from these costs, the costs of the entire population could be determined and divided into subgroups of hemophilia type and severity, thus avoiding overestimation or underestimation of costs and providing a representative estimate of the Spanish sample.

Finally, in relation to unit costs, data from public sources were used for the components related to direct medical costs, but nonmedical costs were reported directly by patients and calculated by pooling publicly available information and internal unit cost databases.

## CONCLUSION

This descriptive analysis provides more information on the economic and humanistic burden of adult patients with moderate and severe HA and HB, without inhibitor diagnosis, in the Spanish setting. The results suggest that, regardless of the type of hemophilia, greater disease severity was associated with increased costs and a concomitant decrease in patient-reported HRQoL. Advances in hemophilia care are challenged to improve patient HRQoL while considering the sustainability of the system. Therefore, the descriptive data from the CHESS II observational study is a source of evidence for the knowledge of patients with a rare disease and useful for new therapies in the field of hemophilia.

## Supplementary Material

Online Supplementary Material
