# Supplementary material for: Economic and Humanistic Burden of Moderate and Severe Hemophilia A and B in Spain: Real-World Evidence Insights from the CHESS II Study
Source: J Health Econ Outcomes Res. 2024 May 6;11(1):122–33. doi: 10.36469/001c.92369 (PMC11078526; doi:10.36469/001c.92369)
Supplement: Online Supplementary Material [file jheor_2024_11_1_92369_226551.pdf]

Journal of Health Economics and Outcomes Research

Online Supplementary Material

Economic and Humanistic Burden of Moderate and Severe Hemophilia A and B in Spain: Real-World Evidence Insights from the CHESS II Study. *JHEOR*. 2024;11(1):122-133. doi:10.36469/jheor.2024.92369

Table S1: Unitary Costs of Resources from Spanish Databases

Table S2. EQ-5D 5L of Patients with Hemophilia A and Hemophilia B in Spain

This supplementary material has been provided by the authors to give readers additional information about their work.

This is an open-access article distributed under the terms of the Creative Commons Attribution 4.0 International License (CCBY-4.0). View this license's legal deed at http://creativecommons.org/licenses/by/4.0 and legal code at http://creativecommons.org/licenses/by/4.0/legalcode for more information.

Table S1. Unitary Costs of Resources from Spanish Databases

**Direct Costs**	**Unit Cost (€, 2022)**[a]
Specialist consultations (per consultation)[26]	
Dentistry	49.52
Diet and nutritional support	141.26
Emergency medicine and acute surgery	176.98
General practice	37.49
General surgery	134.39
Genetics	190.45
Hematologist	169.79
Hepatology	198.91
Immunology	207.55
Infectious diseases	122.31
Nurse specialist	19.42
Orthopedics	160.22
Pain management	140.88
Physiatry/rehabilitation	19.80
Physiotherapy	19.80
Psychiatry	145.73
Psychology	55.31
Rheumatology	173.22
Lab tests (per unit)[26]	
Biochemistry blood test	21.02
CD4 count	52.45
Chromogenic factor assay	62.55
Coagulation test	15.22
Computed tomography	212.63
Hemoglobin	18.59
Hepatitis C virus antibody	37.72
Human immunodeficiency virus antibody	14.66
Magnetic resonance imaging	318.31
Mix test	240.33
One-stage factor assay	43.00
Ultrasonography	81.95
Urinalysis	11.34
X-ray	127.69
Hospitalizations[26]	
Cost per hospital ward day	832.99
Cost per day case	169.79
Cost per ICU day	1180.73
Joint procedures[26]	
Arthrocentesis	279.35
Arthrodesis	2619.84
Arthroplasty	809.052
Arthroscopy	2837.26
Synovectomy	2710.48

Table S1. Unitary Costs of Resources from Spanish Databases

Health devices[27]	
Brace	22.62
Cane	8.19
Crutches	7.79
Wheelchair	145.64
Home alterations[27]	
Ramp	237.09
Stairlift	6174.25
Walk-in shower	304.83
Alternative complementary therapies[27]	
Chiropractor (private)	44.03
Massage/acupuncture (private)	40.47
Nutritionist (private)	62.10
Occupational therapist	56.45
Reflexology	33.87
Swimming/aerobics	3.95
Physiotherapist (private)	101.53
Psychologist (private)	45.16
Yoga/Pilates	11.29
Formal care, per hour[27]	33.87
Transport	
Fuel (per km)[19]	0.39
**Treatments authorized in Spain[24,25]**	**Cost per UI (€, 2022)**
EHL FVIII	0.52
SHL FVIII	0.52
Plasma-derived FVIII	0.39
EHL FIX	1.48
SHL FIX	0.42
Plasma-derived FIX	0.49
**Indirect costs**	**Unit cost (€, 2022)**
Wage (informal caregiver), per hour[19]	7.82
Wage (employee), per hour[b]	16.35

[a]Adjusted to December 2022.
[b]Based on minimum interprofessional wage in Spain.

Table S2. EQ-5D 5L of Patients with Hemophilia A and Hemophilia B in Spain

**Parameter**	**Hemophilia A (HA)**			**Hemophilia B (HB)**		
	**Moderate (n=66)**	**Severe (n=115)**	**Moderate and Severe (n=181)**	**Moderate (n=28)**	**Severe (n=79)**	**Moderate and Severe (n=107)**
EQ-5D-5L index score, mean (SD)	0.81 (0.15)	0.77 (0.18)	0.78 (0.17)	0.86 (0.17)	0.70 (0.22)	0.73 (0.22)
EQ-5D-5L–specific domains						
Anxiety/depression dimension	1.61 (0.50)	1.93 (0.93)	1.84 (0.84)	1.70 (1.25)	1.84 (0.85)	1.82 (0.92)
Pain dimension	1.82 (0.72)	2.09 (0.97)	2.01 (0.91)	1.60 (0.70)	2.33 (0.77)	2.20 (0.80)
Selfcare dimension	1.29 (0.53)	1.46 (0.70)	1.41 (0.66)	1.20 (0.42)	2.05 (1.01)	1.89 (0.98)
Mobility dimension	1.68 (0.72)	1.68 (0.80)	1.68 (0.77)	1.40 (0.70)	2.22 (1.18)	2.07 (1.15)
Usual activity dimension	1.43 (0.63)	1.71 (0.75)	1.63 (0.73)	1.40 (0.52)	2.16 (1.07)	2.02 (1.03)
EQ-VAS	74.14 (13.75)	65.31 (15.30)	68.06 (15.32)	75.64 (12.92)	69.71 (16.93)	70.88 (16.29)
Patient self-reported satisfaction						
Patient satisfaction with healthcare received (graphic scale 1-10)	8.42 (1.17)	8.15 (1.22)	8.22 (1.21)	8.36 (1.12)	8.07 (1.32)	8.13 (1.28)

Abbreviations: EQ-VAS, EuroQol visual analog scale.
